# Papaya latex mediated synthesis of prism shaped proteolytic gold nanozymes

**DOI:** 10.1038/s41598-023-32409-7

**Published:** 2023-04-12

**Authors:** Ajoy Kumar Das, Jon Jyoti Kalita, Maina Borah, Suradip Das, Manav Sharma, Dhiren Saharia, Kushal Konwar Sarma, Samrat Bora, Utpal Bora

**Affiliations:** 1 Department of Botany, Arya Vidyapeeth College, Gopinath Nagar, Guwahati, Assam 781016 India; 2 Department of Biosciences and Bioengineering, Indian Institute of Technology, Guwahati, Assam India; 3 Department of Botany, Pandu College, Pandu, Guwahati, Assam 781012 India; 4 Saharia’s Path Lab and Blood Bank, Guwahati, Assam 781 005 India; 5 Department of Surgery and Radiology, College of Veterinary Sciences, Assam Agriculture University Campus, Khanapara, Guwahati, Assam 781 022 India

**Keywords:** Biochemistry, Biological techniques, Chemistry, Materials science, Nanoscience and technology

## Abstract

Beyond natural enzymes, the artificially synthesized nanozymes have attracted a significant interest as it can overcome the limitations of the former. Here, we report synthesis of shape controlled nanozymes showing proteolytic activity using *Carica papaya* L. (papaya) latex. The nanozymes synthesized under optimized reaction conditions exhibited sharp SPR peak around 550 nm with high abundance (45.85%) of prism shaped particles. FTIR analysis and coagulation test indicated the presence of papaya latex enzymes as capping agents over the gold nanoprisms. The milk clot assay and the inhibition test with egg white confirmed the proteolytic activity of the nanozymes and the presence of cysteine protease on it, respectively. The nanozymes were found to be biocompatible and did not elicit any toxic response in both in-vitro and in-vivo study. Based on our findings, we envisage that these biocompatible, shape-specific nanozymes can have potential theragnostic applications.

## Introduction

Enzymes which are present in biological systems are exquisite catalyst and accelerate every biological process with high degree of efficiency and specificity^1^. However, ex-situ application of such biologically derived enzymes is challenged by their instability, low selectivity, sensitivity to environmental condition and high cost in preparation and purification^1^. Hence, there is a need of developing appropriate platforms for improving the functionality of biologically derived enzymes which would facilitate their use in biomedical, pharmaceutical and industrial applications. Attempts have been made to synthesize nanozymes by conjugating such enzymes with several metallic nanoparticles like Ag^2^, Pt^3^ as well as polymeric nanoparticles^4^. Along with that, plant derived enzymes (mostly collected from latex) have also been used for the synthesis, capping and stabilization of gold nanoparticles (GNPs)^5–7^. However, such methods exhibit a high degree of heterogeneity in terms of shape, size and surface area.

Moreover, compared to isotropic nanoparticles (example, sphere), anisotropic nanoparticles are more advantageous as optoelectronic properties can be tuned due to their geometry^8^. Thus, the shape controlled anisotropic nanoparticles can increase the target specificity and improve its release kinetics^9,10^. Owing to these advantages, a myriad of nanomorphologies ranging from rods, wires to prisms, stars, flowers etc. have been synthesized for various purposes. Exhibition of strong surface plasmon absorption in the near-infra-red region makes nanorods a preferable material in diagnosis, tumor imaging and therapeutic application^11,12^. Apart from nanorods, gold nanoprisms have also attracted a tremendous research attention as they enjoy excellent surface enhanced Raman signals as well as plasmonic features both in visible and IR region with well defined crystallographic facets^13^. These features make gold nanoprism a promising candidate in the field of biosensing and biomedicine. Recently, such particles are used in detecting ions, molecules as well as nucleic acid and aptamer sensing. It has also been used in curing certain neurodegenerative diseases and as a cell starving agent in cancer treatment^14^.

Although, structurally specific nanoparticles are routinely synthesized using physical and chemical methods but challenges like storage, functionality as well as stability, long term toxicology are yet to be fulfilled^15^. Few limited reports are found to be available about shape controlled synthesis of nanozymes. In the present study, we have attempted to synthesize prism shaped gold nanozymes utilizing *Carica papaya* L. latex as reducing agent. The synthesized nanozymes was further characterized for their physico-chemical properties. Further, the toxicity of the synthesized prismatic nanozyme was verified by both in-vitro and in-vivo study.

## Results

### Preparation of aqueous fraction and synthesis of gold nanozymes

Aqueous fraction (AF) obtained by centrifugation of the collected papaya latex was used for the synthesis of prismatic gold nanozymes. The concentration of protein present in AF was estimated with Bradford standard curve and was found to be 7.4 µg/ml.

The synthesis of prismatic gold nanozymes was done by optimizing different reaction parameters such as concentration of AF, HAuCl_4_ and reaction time using UV–VIS spectrophotometer. The optimum condition was found to be 5% of AF (v/v) and 0.75 mM HAuCl_4_ with 50 s reaction time (Table 1).

**Table 1:** Optimization of reaction parameters for the synthesis of prism shaped nanozymes.

Reaction parameters	Characteristics of SPR peak
Concentration of AF (%)	
1	No peak, unable to reduce Au^3+^
2	No peak, unable to reduce Au^3+^
3	No peak, unable to reduce Au^3+^
4	Less and broad peak, partial reduction of Au^3+^
**5**	**Intense and narrow peak, complete reduction of Au**^**3+**^
6	Negligible change in intensity
7	Negligible change in intensity
8	Red shifting
9	Red shifting
10	Red shifting
Concentrationof HAuCl_4_ (mM)	
0.25	No peak
0.375	No peak
0.50	Less and broad peak
0.625	Less and broad peak
**0.750 **	**Intense and narrow peak**
0.875	No significant gain in intensity
1.000	No significant gain in intensity
1.125	Red shifting
1.250	Red shifting
Reaction time (s)	
30	No peak
35	No peak
40	Less and broad peak
45	Enhancement in intensity
**50**	**Intense and narrow peak**
55	Negligible change in intensity
60	Negligible change in intensity
65	Red shifting
70	Red shifting

The optimized reaction parameters are in [bold].

### Characterization of synthesized gold nanozymes

**Figure 1:**
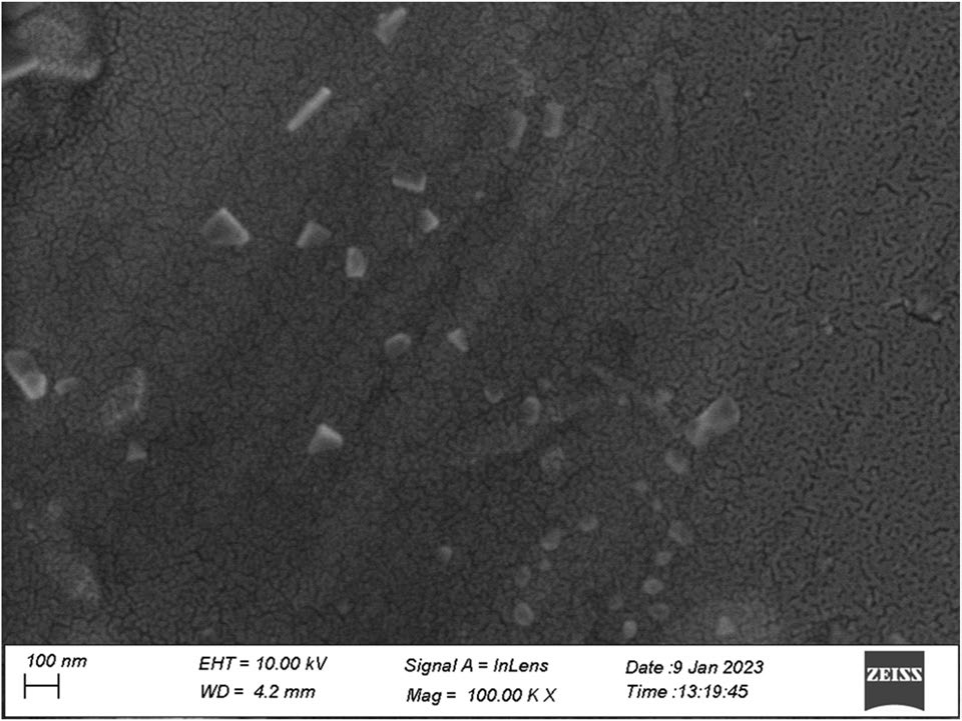
SEM micrograph of gold nanoparticles.

**Figure 2:**
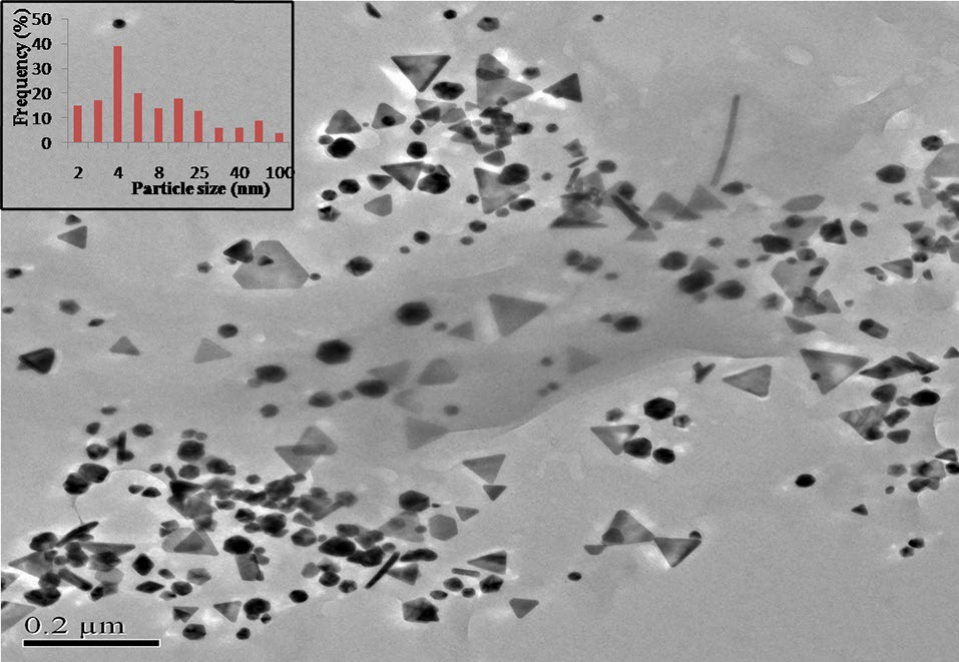
TEM micrograph of gold nanoparticles, inset: size distribution histogram.

**Figure 3:**
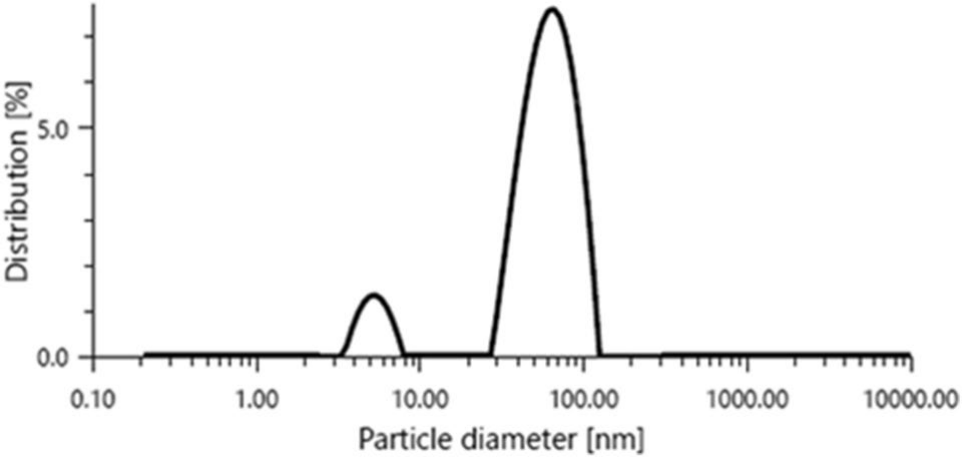
DLS graph showing size distribution of the nanoparticles.

**Figure 4:**
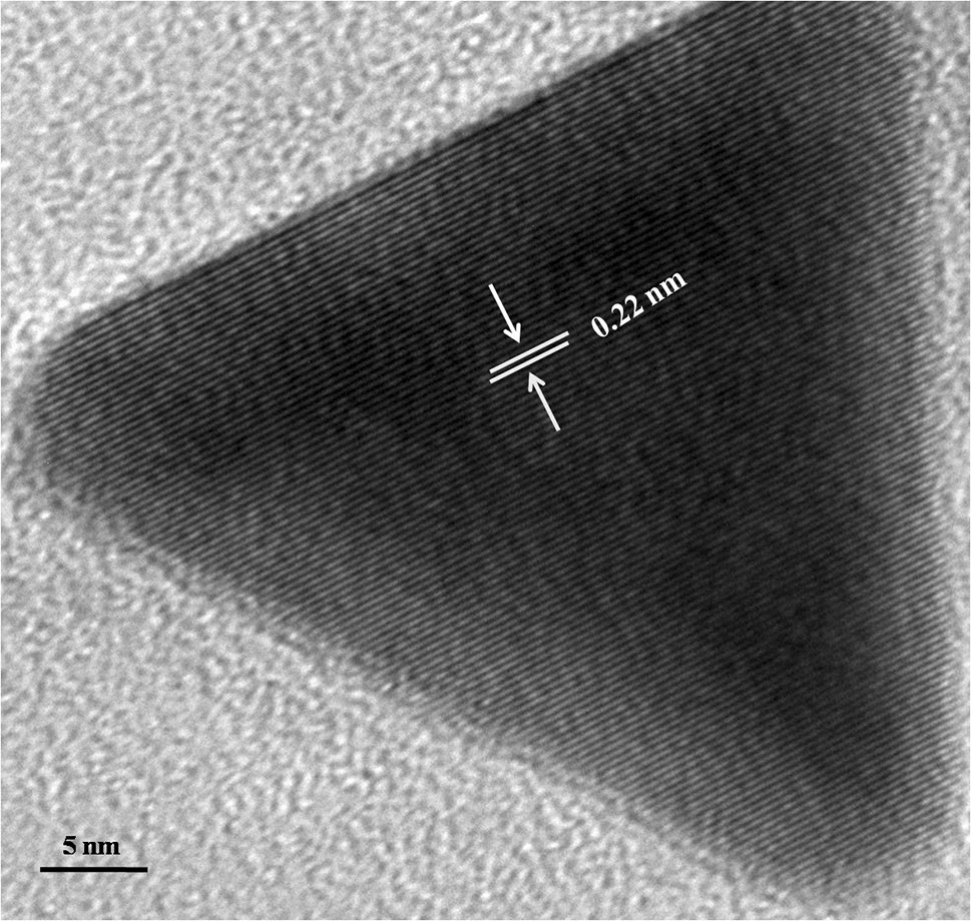
HRTEM image of prismatic gold nanozymes.

The size and shape of the gold nanozymes synthesized with optimized reaction condition were observed by Scanning Electron Microscope (SEM) and Transmission Electron Microscope (TEM) analysis. Both the SEM and TEM image clearly revealed the dominance of prism shaped nanozymes (Figs. 1, 2). A histogram corresponding to TEM image was prepared representing the size distribution of the prismatic nanozymes (Fig. 1). The size variation of the prismatic nanozymes was found to be ranged from 2 to 100 nm. The average size was found to be 14.6 ± 3.045 nm. The percentage of prismatic nanozymes of total population was found to be 45.85%. A very less number of rod like and hexagonal particles were observed in the TEM image. The edge length (l) and the thickness (t) of the synthesized prismatic nanozymes were found to be ranged from 2 to100 nm and from 0.5 to 25 nm respectively. The aspect ratio (l/t) estimated was 4. The surface area of the interface was ranged from 140 to 5000 nm^2^. For maximum prismatic nanozymes, the surface area of the interface was found to be 4000 nm^2^. Height of the synthesized prismatic nanozymes ranged from 15.5 to 86.6 nm which was calculated by using the formula (l) sin60. The size distribution was also determined by DLS method (Fig. 3). The DLS peak showed size variation ranges from 5 to 100 nm (Fig. 3). UHRTEM (Ultra High Resolution TEM) revealed clear lattice fringes of 0.22 nm (Fig. 4). Selected area electron diffraction pattern (SAED) of a single prismatic particle confirmed the crystalline nature of the synthesized nanoprisms by observing three Debye–Scherrer’s rings corresponding to (1 1 1), (2 0 0) and (2 2 0) gold crystalline planes (Fig. 4).

**Figure 5:**
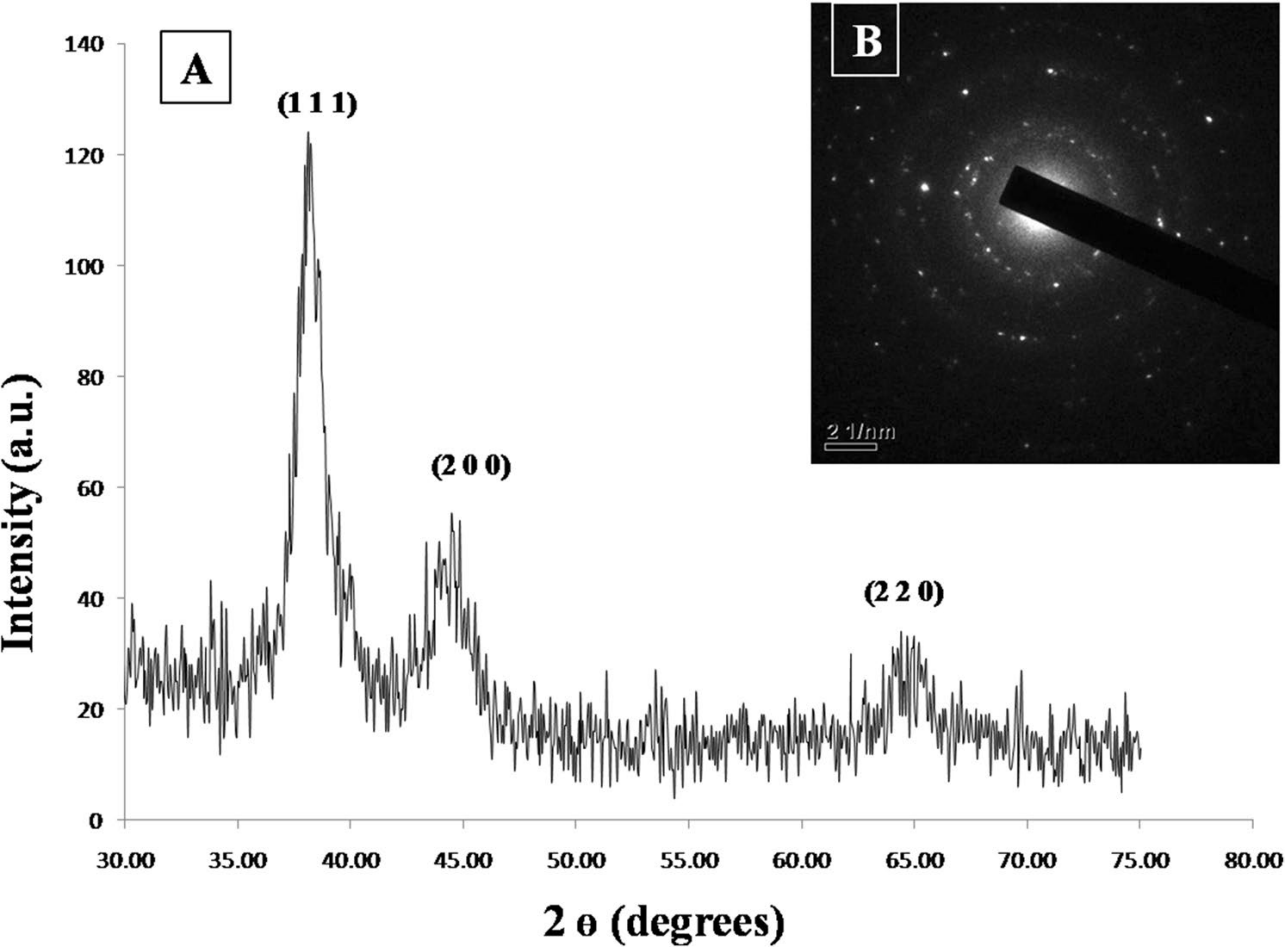
XRD patterns of synthesized prismatic gold nanozymes with optimized condition, inset: SAED pattern.

The crystalline nature of the synthesized prismatic gold nanozymes was confirmed by the X-Ray Diffraction (XRD) analysis. The XRD spectrum exhibited three peaks at 2Ѳ values of 38.2°, 44.36° and 64.76° respectively (Fig. 5).

**Figure 6:**
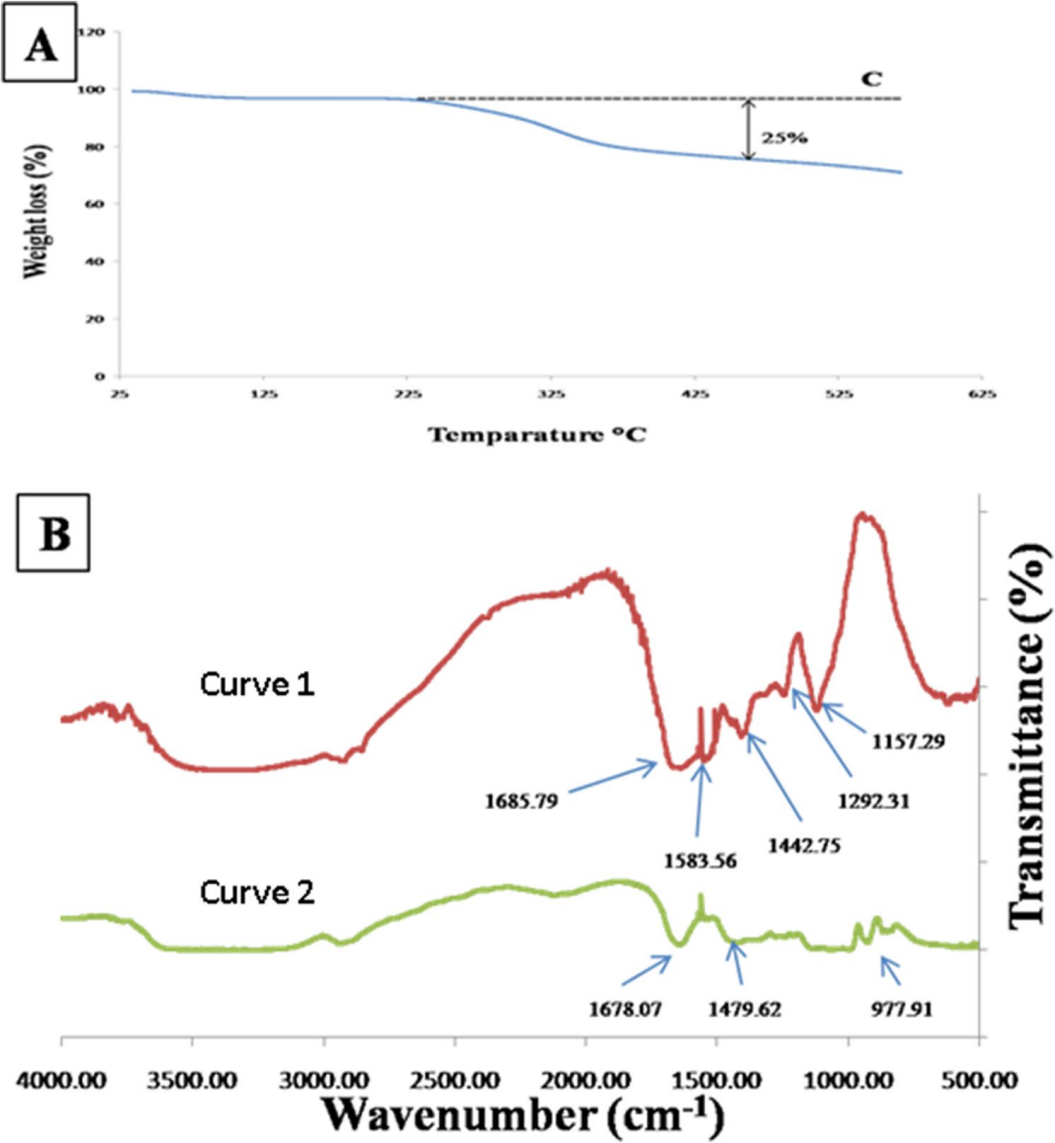
(**A**) TGA spectra of the synthesized prismatic gold nanozymes where C is the baseline (**B**) FTIR spectra of aqueous fraction (AF) of *Carica papaya* L. (curve 1) latex and synthesized gold nanozymes with 1 mM HAuCl4 (curve 2).

The capping of gold nanoprisms by latex protein was investigated by FTIR spectra, coagulation test and Thermo gravimetric analyzer (TGA). Coagulation test revealed no change in colour of the nanozyme solution. TGA spectra of prismatic nanozymes occurred at a wide range of temperature (Fig. 6A). Analysis of FTIR spectrum obtained for lyophilized AF revealed strong IR bands at 1685.79 cm^−1^, 1442.75, 1583.56 and 1292.31 cm^−1^ (Fig. 5B, curve 1)and that of prismatic nanozymes showed bands at 1678.07 cm^−1^, 1479.62 cm^−1^ and 1020 cm^−1^ (Fig. 6B, curve 2).

### Confirmation of proteolytic activity of prismatic gold nanozymes

**Figure 7:**
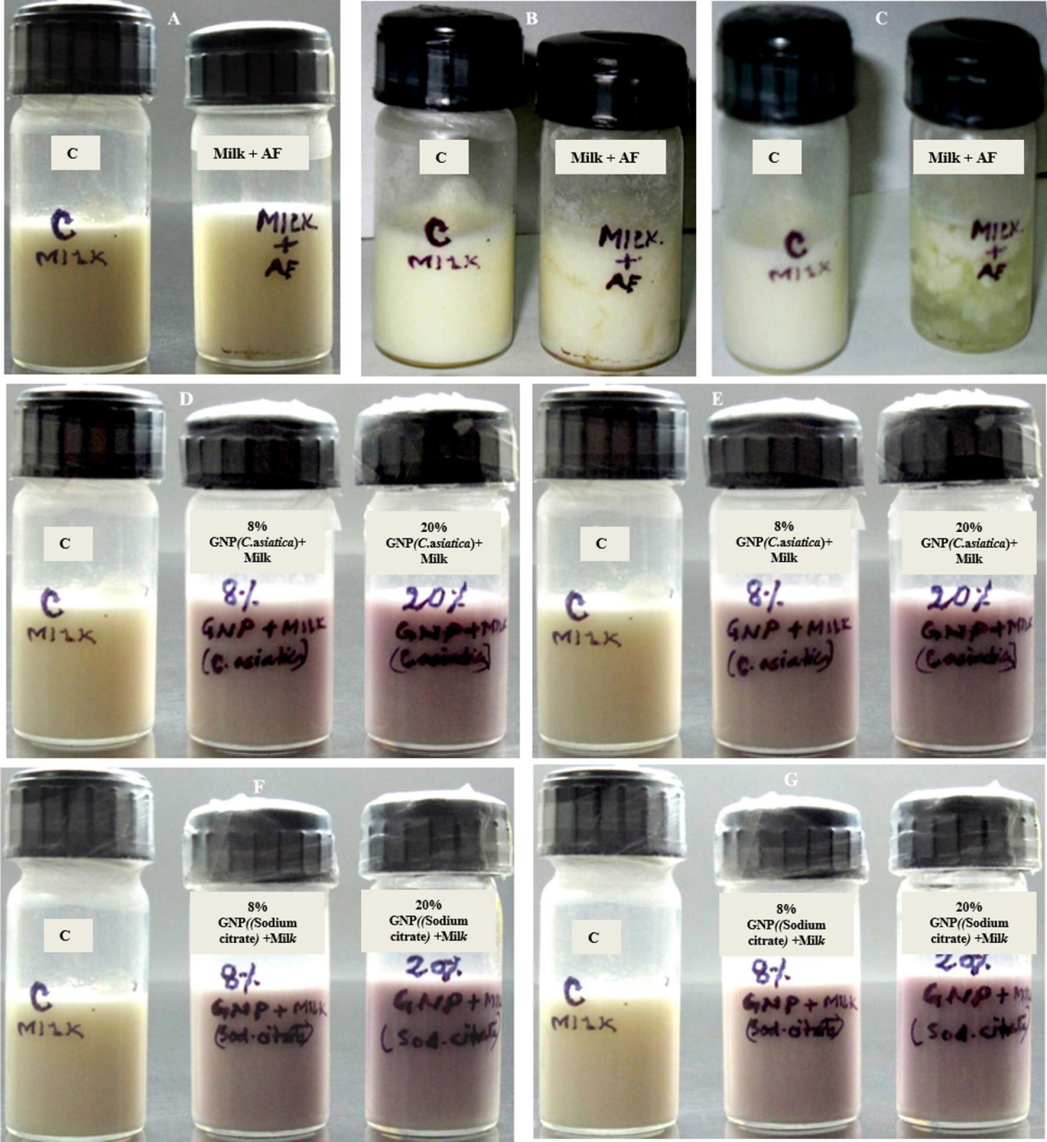
Milk clot assay (**A**–**C**) with milk + AF, **A** = 0 h, **B** = ½ h, **C** = 1 h (**D**–**E**) with milk + GNPs synthesized with *Centella asiatica*, **D** = 0 h, **E** = 72 h. (**F**,**G**) with milk + GNPs synthesized with sodium citrate, **F** = 0 h, **G** = 72 h.

**Figure 8:**
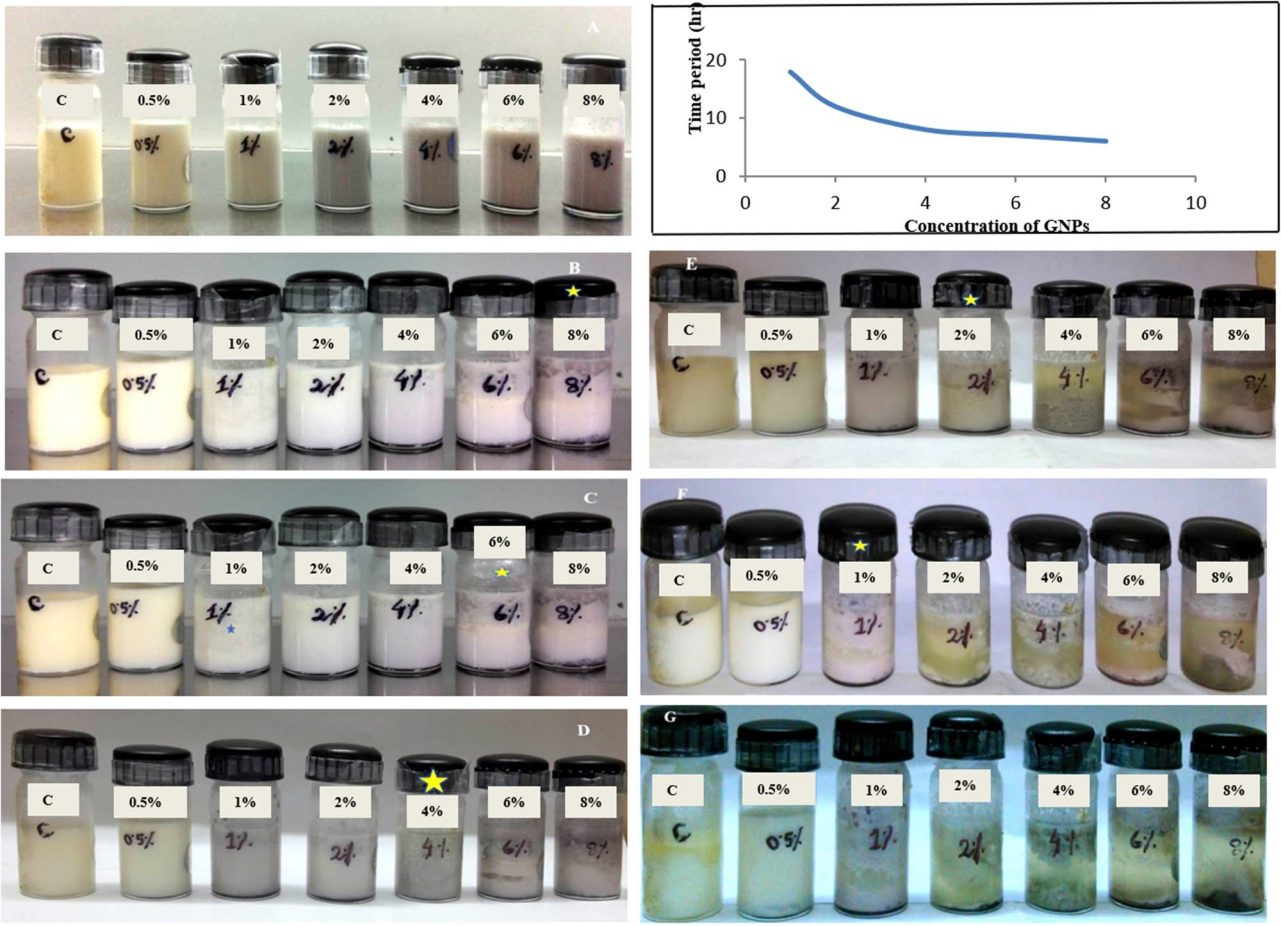
Milk clot assay (**A**–**G**) with different concentrations of prismatic gold nanozymes (0.5, 1, 2, 4, 6 and 8%) upto 72 h with images taken at initiation of curdling; inset: histograms showing initiation of curdling.

**Figure 9:**
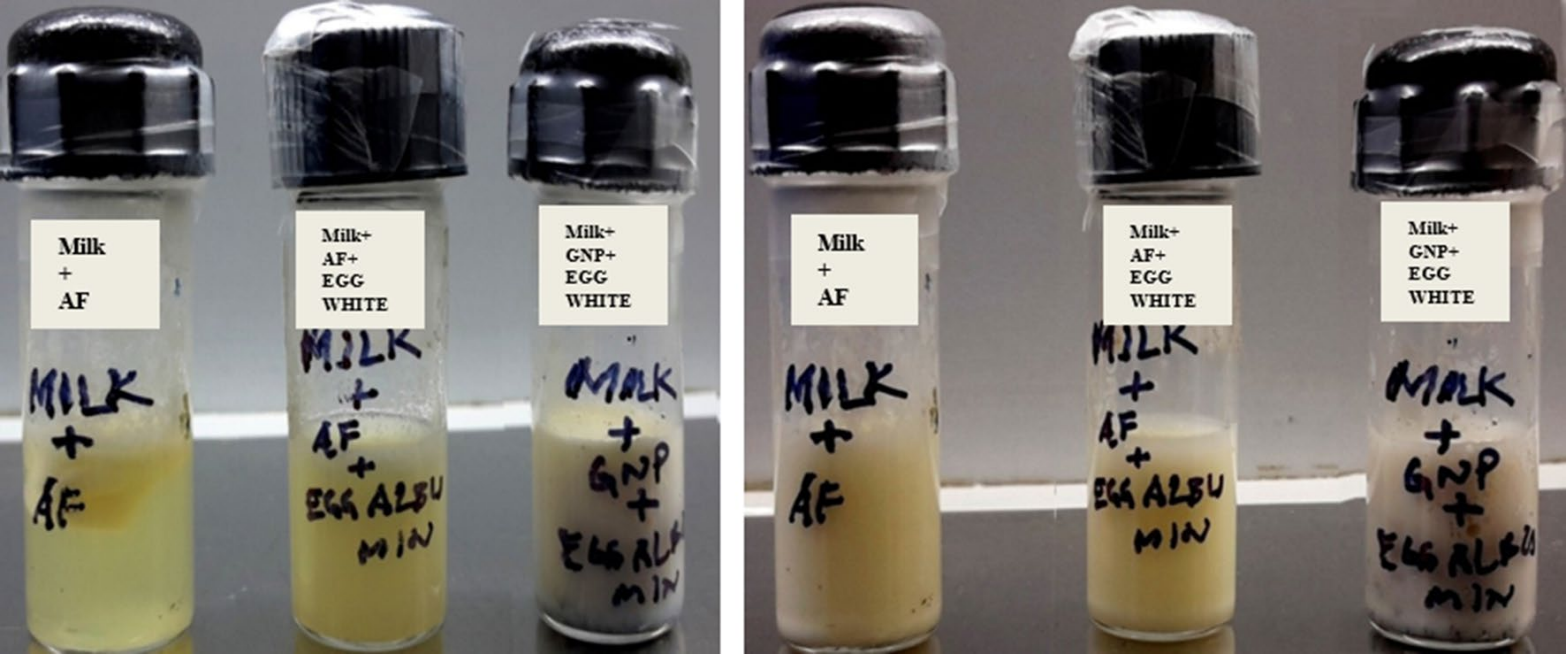
Papain inhibition test (**A**,**B**) with (milk + chicken egg white), (milk + chicken egg white + prismatic gold nanozymes), A = 0 h., B = 72 h^.^

The enzymatic functionality of the synthesized prismatic gold nanozymes was confirmed by milk clot assay (Figs. 7, 8) and papain inhibition test using chicken egg white (Fig. 9). Clotting was also observed for the mixture of AF and sterile milk. Sterilized milk, GNPs synthesized by sodium citrate and *Centella asiatica* was used as control group for this assay (Fig. 7). In the milk clot milk clot assay involving the mixture of AF and sterile milk, clotting was observed within 30 min (Fig. 7). Different concentrations of nanoprisms (0.5–8%) mixed with sterilized milk in the ratio of 1:10 was used for milk clot assay (Fig. 8). For the mixture of nanoprisms and milk, clotting formation was initiated within 6 h with 8% concentration of nanoprisms and curd was formed within 7 h For 6%, 4% and 2% concentration, the initiation of clotting was found to be observed within 7 h, 8 h and 12 h, respectively (Fig. 8). For these concentrations, the curd was found to be formed within 8 h, 12 h and 18 h, respectively. The minimum concentration of nanozymes for clotting to be observed was found at 1% and which took 18 h to initiate the formation of curd. No clotting was found to be observed below 1% of GNPs though the reaction was allowed to run up to 72 h (Fig. 8). The enzymatic activity (Milk clotting activity) was found varied for each studied concentration. Reaction mixtures of AF, sterilized milk and synthesized nanoprisms prepared with chicken egg white in the ratio of 1: 1 was used for papain inhibition test (Fig. 9). The papain inhibition test revealed no clotting of milk for any of the reaction mixture (Fig. 9). Clotting of milk was not found to be formed in case of control group.

### In-vivo and in vitro toxicity study of synthesized nanozymes

**Figure 10:**
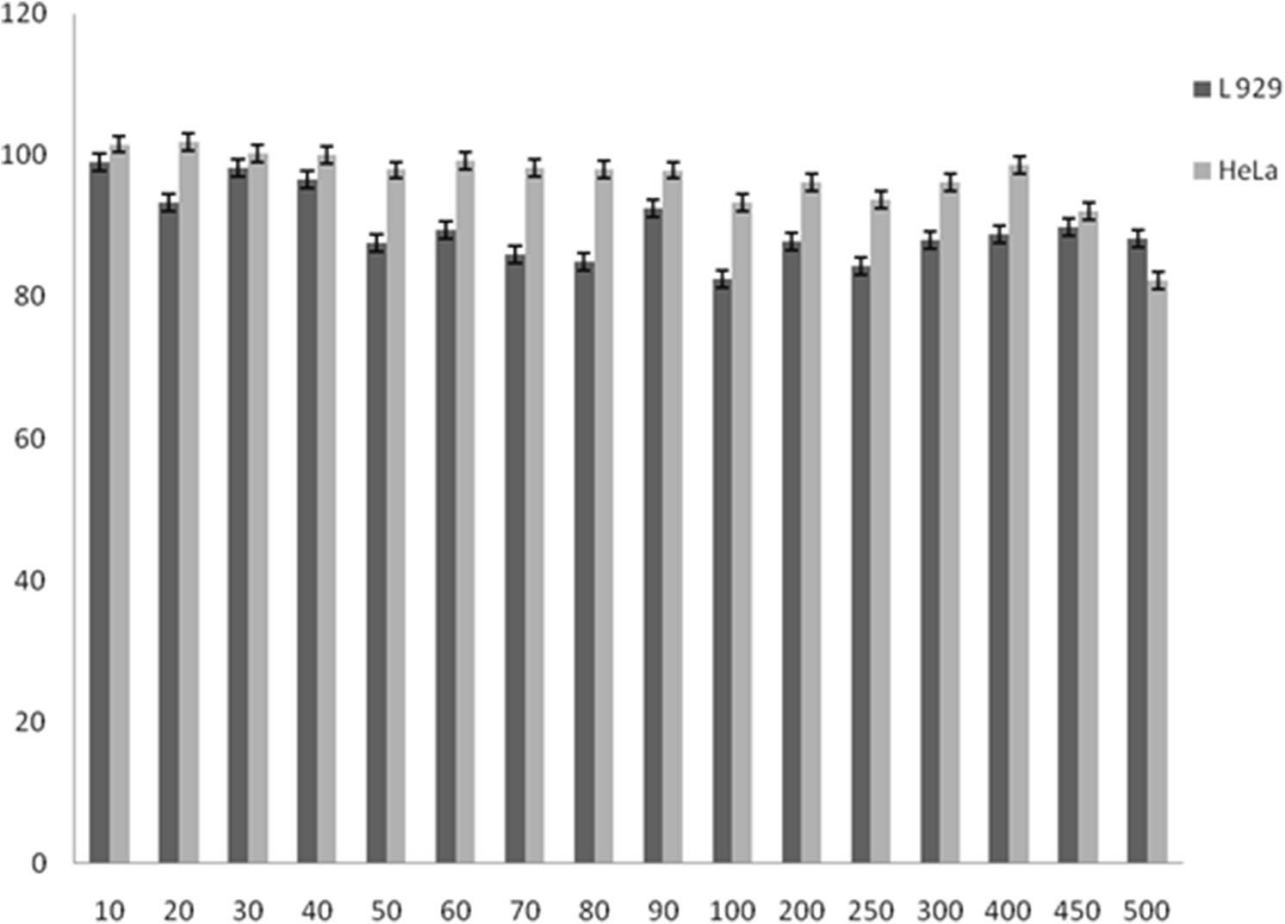
Cytotoxicity of synthesized prismatic gold nanozymes.

The cytotoxicity of the synthesized nanozymes was studied using L929 and HeLa cell lines. The MTT assay revealed no cytotoxic effects after treatment of cell lines for 24 h. Cells of the both cell lines showed high viabilities (89 ± 4% and 96 ± 2.3% respectively) even up to 500 µM concentration of nanozymes (Fig. 10).

**Figure 11:**
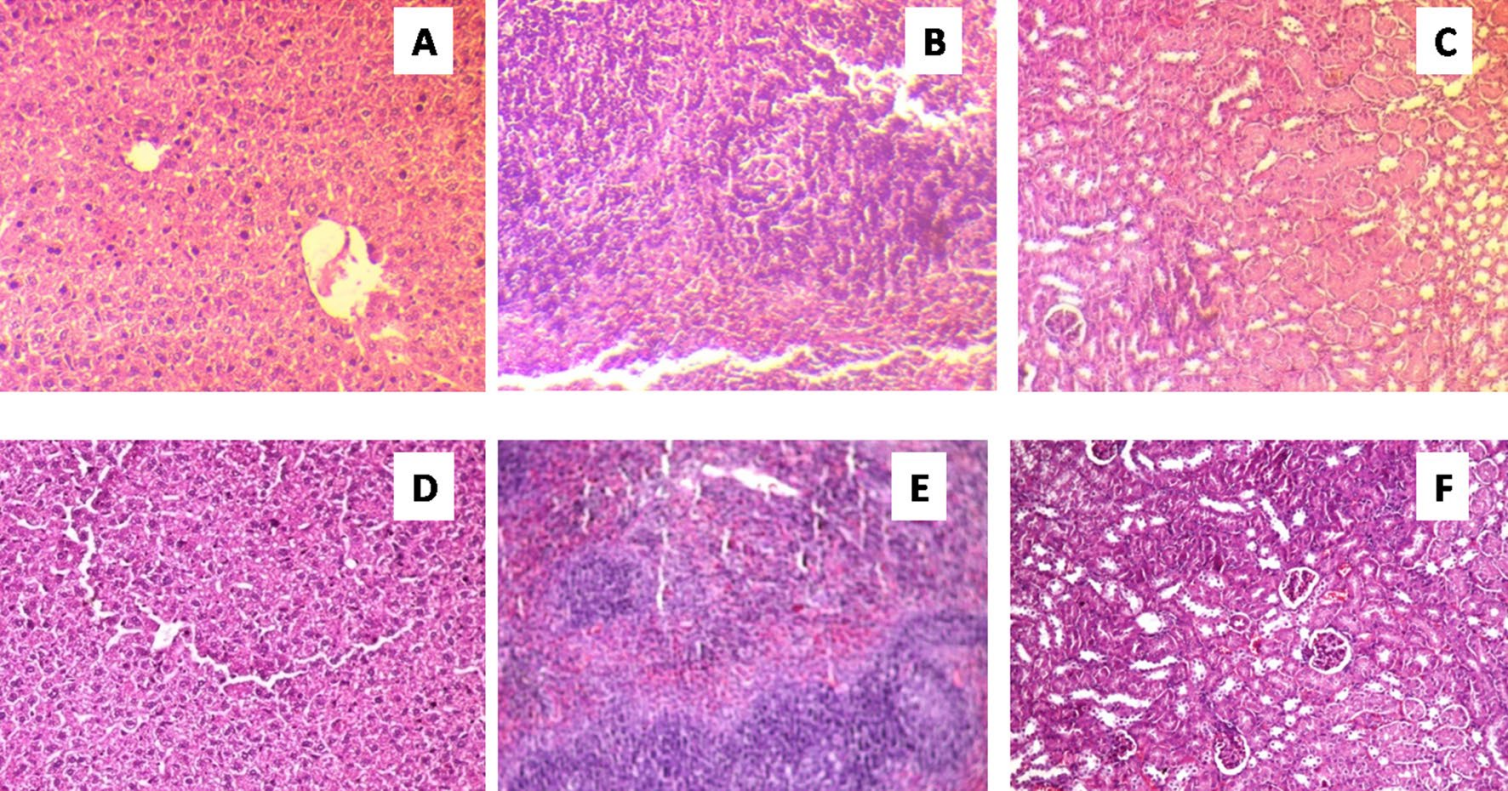
Histology of Liver, spleen and kidney (**A**–**C**) = normal rat (control); (**D**–**F**) nanozynme treated mice.

**Figure 12:**
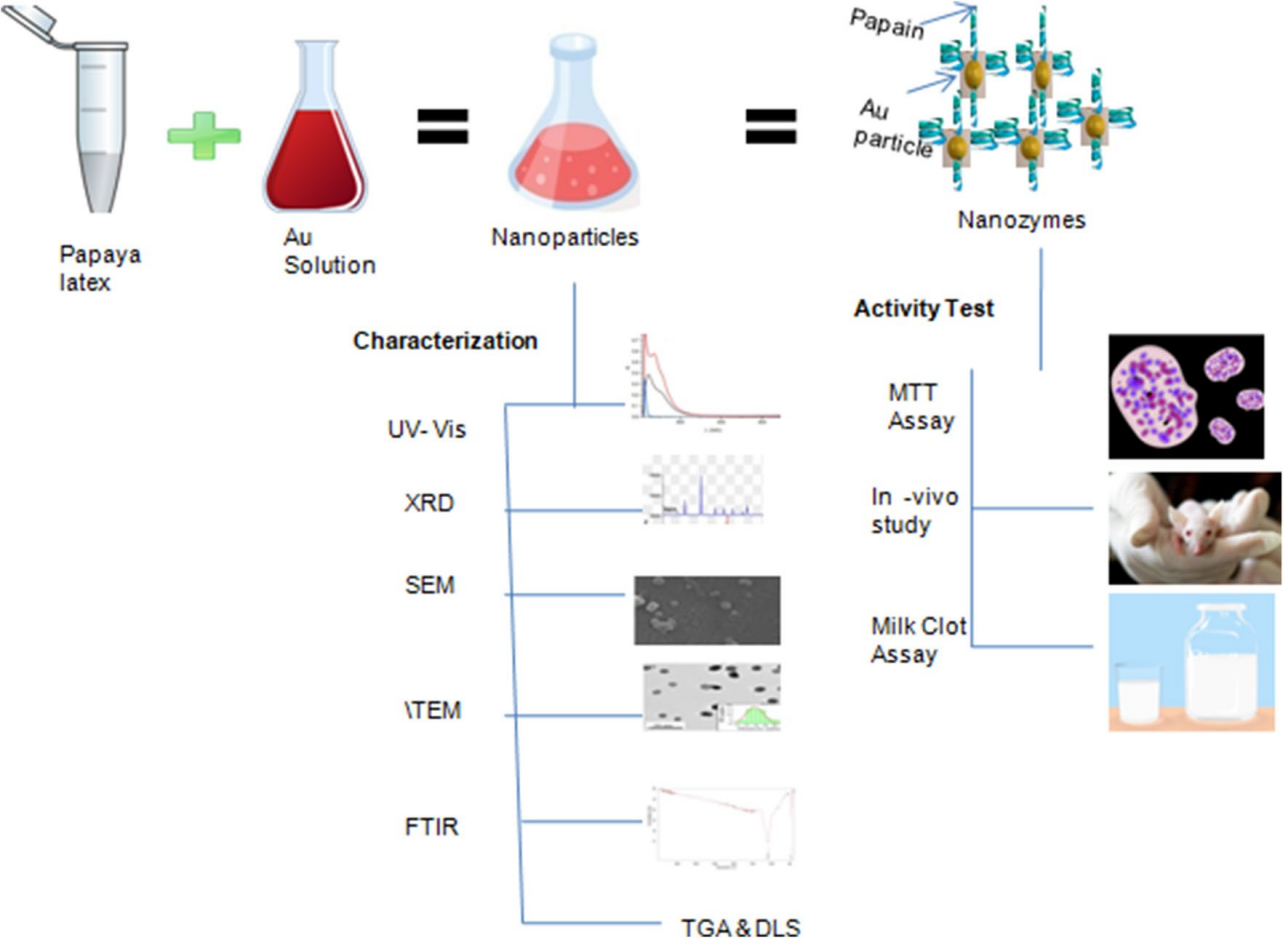
Schematic representation of synthesis of nanoparticles, its characterization (UV–VIS, XRD, SEM, TEM, FTIR, TGA, DLS) and activity tests (MTT test, in-vivo study and Milk Clot Assay).

Acute oral toxicity of synthesized prismatic gold nanozymes was studied by using swiss albino female mice. The animals were split into four groups according to oral dose with a range of 2000 mg (Group 1), 1000 mg (Group 2), 300 mg (Group 3) and 0 mg (Group 4, control) per kg BWt, respectively. In this study, physiological and behavioral changes in the animals for a period of two weeks were observed. No clinical abnormalities were observed in any of the groups studied. Gross examination of all vital organs revealed normal study. Histological findings were found to be normal in all groups (Fig. 11). However, group (with highest dose @ 2000 mg per kg BWt) revealed mild sinusoidal congestion in spleen and mild cloudy swelling of liver with intact follicular morphology and overall normal cellular architecture for spleen and liver, respectively (Fig. 11). In spite of mild histological aberrations in liver and spleen, no neurological and gastrointestinal abnormalities were detected in this dosing group (Fig. 11). The whole mechanism starting from synthesis of nanoparticles to the milk clot assay has been presented in a schematic diagram (Fig. 12).

## Discussion

The shape of nanoparticles plays an important role in determining its properties hence an inclusive knowledge over shape is very essential in developing nanodevices with specific function^8,63^. Therefore, systematic efforts have been made for the synthesis of shape controlled nanoparticles. The synthesis of anisotropic nanoparticles has attracted more interest due to its superiority to isotropic (spherical) nanoparticles in terms of physiochemical properties^16^. The spherical nanoparticles can easily be synthesized by reduction of the metal salt as spheres possess lowest energy^17^. However, the synthesis of anisotropic nanoparticles is challenging and requires efficient protocol^18–20^. Anisotropic nanopartcles can be synthesized by controlling the reaction parameters such as concentration of metal ion, reducing agent, stabilizing agent and reaction time though the exact working mechanism of these parameters is yet to be understood^8,20^. The nanoprisms are considered as one of the recent members in the list of anisotropic nanomaterials^21^. As it exhibits attractive quadruple plasmon properties, it draws enormous interest upon its synthesis and application^22^. But available method for the synthesis of nanoprisms is rather scanty^63^. However, a very few approaches have been reported for the synthesis of functional nanoprisms using biogenic extract. Here, we have used papaya latex for successful synthesis of prism shaped gold nanozymes. The formation of gold nanoprisms was confirmed by UV–VIS spectral analysis. The reaction condition for the synthesis of prismatic gold nanozymes was optimized on the basis of characteristic plasmon bands exhibited by concentration of AF, HAuCl_4_ and reaction time (Table 1).

The physical nature of the synthesized particles was found to be prism shaped as revealed by SEM and TEM image (Figs. 1, 2). The percentage of prism shaped nanoparticles was found as 45.85% which is higher than the earlier reports. However, the DLS peak revealed a size distribution of the nanoparticles which was ranged from 5 to 100 nm (Fig. 3). For the synthesis of anisotropic gold nanoparticles, it needs selective capping agent during growth as it possesses symmetric face centered cubic (fcc) lattice^23,24^. It is reported that iodide (I) ion facilitated the formation of gold nanoprisms by preferential binding to (111) facet of the nanocrystal^25^. Cetyltrimethylammonium bromide (CTAB) that act as shape directing agent also enhances the synthesis of triangular prisms^26^. The aldoses (reducing sugar) present in the lemongrass has also been reported for the synthesis of nanoprisms by reducing Au+^8^. In the current study, the enzymes present in the latex that were used as reducing as well as capping agent has the capability to form gold nanoprisms in optimum reaction condition. Moreover, adsorption of capping agent dramatically effects the free surface energy of various crystal faces of nanoparticles^27^. Since the ratio of free surface energy and total energy is one of the structures determining factor the adsorption of capping agent is crucial in synthesis of specific shaped nanoparticles^28^. It is assumed that the biomolecules present in the latex chemisorb preferentially on the (111) plane of the nanocrystals and thereby lower down the free surface energies of (111) surfaces which results into the formation of (111) facets and the nanoprisms. The exhibition of clear lattice fringes of 0.22 nm specifies the preferential growth of prisms on the (1 1 1) plane as revealed by UHRTEM (Fig. 4). The interplanner distance of the Au (1 1 1) was in accordance with (1 1 1) d-spacing of bulk Au (0.2355 nm)^15,22^. The appearance of rod like nanoparticles along with prismatic nanozymes might be due to change in orientation of the prisms. The hexagonal prisms observed might be due to tip truncation or rounding of the prismatic nanoparticles^28^. The edge length (l) and the thickness (t) of the synthesized nanoprisms was found to be typical for triangular nanoprisms^29^. The SAED pattern of gold nanoprisms indicates that the entire nanoparticle is nanocrystal in nature^30^. The ring structure revealed by SAED is the characteristic of polycrystalline gold (Fig. 5)^15^. The three prominent Bragg reflections exhibited by XRD analysis was corresponding to (1 1 1), (2 0 0) and (2 2 0) lattice planes indicating that the synthesized nanoparticles were fcc gold (Fig. 5).

The purity and the presence of surface coating of the synthesized nanozymes were also confirmed by TGA analysis. The decomposition curve shows significant weight loss of nanoprisms (Fig. 6A). From the decomposition curve, three components can be identified by looking at oxidation temperature and residual mass (Fig. 6A). The first is the starting of weight loss in the range of 225–300 °C and then the rapid loss in the range of 300–600 °C. The mass that remained after heating beyond this temperature comprised the third component which was the residual mass attributed to the capping agent. The starting of weight loss at higher temperature is the measure of the strength of chemisorption of capping agent on surface of gold nanocrystals. Here, the weight loss estimated from the curve is found to be 25% (Fig. 6A).

The stability, coating and purity of nanoparticles are very essential factors in certain field of nanotechnology such as nanomedical, nanofiltration, nanoelectronic etc.^31^. FTIR is one of the important tools to understand the involvement of functional group in interactions between biomolecules and nanoparticles^32^. In our study, FTIR spectral analysis was performed to identify the biomolecules responsible for capping and stabilization of the synthesized nanozymes. The IR peaks at 1685.79 cm^−1^ (Fig. 6B, curve 1) correspond to bending vibration of amide I. Peaks at 1442.75, 1583.56 and 1292.31 cm^−1^ (Fig. 6B, curve 1)can be assigned to amide II and amide III vibration. FTIR peaks at 1678.07 cm^−1^ (Fig. 4B, curve 2) correspond to amide I. Peaks at 1479.62 cm^−1^ (Fig. 6B, curve 2) can be assigned to amide II and C–N stretch coupled with N–H bending respectively^33,34^. Peaks at 1020 cm^−1^ (Fig. 6B, curve 2) appear from C–O–C symmetric stretching and C–O–H bending^35^. By observing FTIR spectra of both papaya latex powder and synthesized prismatic nanozymes, it is found that there is a shift of amide band from 1685.79 to 1678.07 cm^−1^ (Fig. 6B, curve1 and 2). This suggests possible attribution of (NH) CO group in binding with the particles. Since most of the FTIR peaks are corresponding to bending vibration of amide bonds it indicates that the capping material of the synthesized nanoprisms were the enzymes present in the latex. The synthesized nanozymes were found to be stable after 90 days of storage at 4 °C as evident from UV–VIS spectrum (Supplementary Fig. 3).

In comparison to other coating and stabilizing agent such as polyetheleneglycol (PEG), polyelectrolytes and other ionic and non-ionic polymers, protein coating confers high stability to the nanoparticles^36^. Here, the coagulation test also revealed high colloidal stability of the synthesized nanozymes and well capping of the biomolecules. The sufficient stability and capping of biomolecules might be due to binding of proteins (enzymes) with nanoparticles through strong gold-sulfur bond^37^. The FTIR analysis, TGA analysis and coagulation test clearly suggests that the bioactive molecules originated from latex were well capped with the synthesized nanoprisms thereby making them stable and were found to be pure.

Again, one of the essential criteria for biomedical applications of nanoparticles is surface functionalization^38^. For this reason, apart from synthesis, fabrication of nanoparticle is becoming a fascinating area in the field of nanotechnology especially in biomedical science. The synthesis of artificially fabricated functional nanoparticles showing enzymatic activity (called nanozymes) has attracted significant interest for its promising results in biomedical diagnosis, cancer treatment, immune assay, biosensing, stem cell research, pollution control etc.^1,2^. Specifically in the field of biosensing, such nanozymes immensely help in detecting the analytes with a very high degree because of its signal amplification ability^39^. Due to this property, it can also be used to fabricate cascade sensing system and as well as to develop aptasensors and nanozyme linked immune sorbent assay^40–42^. Moreover, by exploiting the interaction between the nanozymes and analytes, different diagnostic sensors and detection techniques can be adopted for small molecules and ions and heavy metals^43^. The peroxidase mimicking activities of gold nanozymes has widely been used for TMB (Tetramethylbenzidine) oxidation in ELISA techniques^44^. With the help of gold nanozymes, another simple and highly sensitive approach has already been established for detection of H_2_O_2_ and glucose concentration in human cells^14^.

Similarly, the fabricated nanozymes have been playing an important role in the field of biomedicine in recent times. Gold nanozymes are acting as a promising candidate in treatment of several deleterious diseases including Alzheimer and cancer. Application of gold nanozymes coated with polyoxometalate-8peptide reduces ROS and amyloid β protein aggregation as well as Cu accumulation in the brain tissues of Alzheimer patients^45^. Use of encapsulated gold nanozymes with Manganese dioxide, PANAM dendrimer etc. have been considered as one of the smart techniques to treat radiotherapy resistant and drug resistant cancer^46,47^. However, information on shape specific functional nanozymes is very limited. Here, we have synthesized prism shaped nanoparticles showing enzymatic activity (which was confirmed by functionality test) and are termed as nanozymes. The functionality test of the prism shaped nanozymes was performed by milk clot assay and papain inhibition test. The bovine milk used for functionality test of nanozymes is basically composed of four different types of casein proteins viz. αS1, αS2, β, κ^48^. Both α and β casein are responsible for the formation of casein micelles in milk. κ molecule stabilizes the casein micelle and thus protects the milk from coagulation^49,50^. Cysteine protease enzyme (papain) has the ability to hydrolyze the specific peptide linkage formed between phenylalanine and methionine residue (-Phe105-Meth106-) present in N terminal and C terminal region of κ molecule^51^. This results into the release of hydrophilic glycosylated, phosphorylated oligopeptide and hydrophobic para k casein. As para k casein does not have the capacity to stabilize the micelles, accordingly it allows the coagulation of milk to form curd^51^. In case of the milk clot assay, as the clotting of milk was observed for the reaction mixtures of AF and sterilized milk and that of synthesized nanozymes and sterilized milk, it indicates that the enzymes present in both AF and nanozymes were cysteine protease (Fig. 7). The milk clot test for other GNPs synthesized by both chemical (Sodium citrate) and green method using other biomaterial (*Centella asiatica*) were also conducted. No clotting was observed for both cases (Fig. 7). This result suggests that amongst all GNPs, only the papaya latex mediated synthesized nanozymes had the capacity to clot the sterilized milk. In this study, the minimum time required for initiation of curdling was found to be 6 h. with 8% nanozymes and the minimum concentration of nanozymes having the capacity to initiate curdling was found to be 1% (Fig. 8). The milk clotting activity (MCA) value of the nanozymes was found to be ranged from 5.208 (U/g) to 14.705 (U/g) for 8 to 1% concentration of nanozymes used for milk clotting.

The milk clot assay clearly revealed that the synthesized nanozymes were functional and proteolytic in nature. The presence as well as the functionality of cysteine protease in both AF and synthesized nanozymes were also confirmed with the help of chicken egg white where cystatin is present naturally (papain inhibition test, Fig. 9). Cystatin (an analogue of human cystatin C) inhibits the activity of cysteine protease through hydrophobic interaction of its binding region with the corresponding binding pockets of the enzyme^52^. The N terminal fragment of cystatin having 11 amino acids also involve in the inhibition process by interacting with the binding pockets of cysteine protease^53,54^. The involvement of N terminal fragment was confirmed by three dimensional structure analysis of cystatin^52^. The papain inhibition test revealed no milk clotting for any of the reaction mixture with egg white cystatin (Fig. 9). In fact, the egg white cystatin inhibited the activity of cysteine protease and thereby milk clotting was not observed. Since cystatin inhibits the function of papain, a cysteine protease, it conclusively unveiled that the capping moiety of the synthesized nanozymes was nothing but papain.

Again, due to promise in biomedical applications, enormous interest has been grown for the production of different types of nanomaterials including GNPs^55,56^. However, addressing of toxicity and health impact is very essential before the use of such materials in real clinical settings^57^. Therefore, it is crucial to study both in vitro and in vivo toxicity of the nanomaterials to be used in biomedical applications. Majority of the reports that investigated the toxicity of nanomaterials are found to be limited to performing only in vitro study^58^. The present study has included both in vitro and in vivo toxicity studies of the synthesized prismatic gold nanozymes. Both the MTT assay (Fig. 10) and in vivo study (Fig.  11) revealed no cytotoxicity and clinical abnormality respectively and thus the synthesized prismatic gold nanozymes were found to be biocompatible.

## Conclusions

We have successfully synthesized higher percentage of prism shaped gold nanozymes. The use of papaya latex as reducing agent for synthesis of nanozymes was found to be stable and non-toxic as evident from both in-vitro and in-vivo study. The prismatic gold nanozymes thus synthesized was found to have functional activity. The synthesized nanozymes due to its shape specificity and proteolytic activity might be exploited in the field of biosensing, cell imaging, target specific drug delivery, therapy, catalysis and photonics etc. In addition, as the nanoprisms are fabricated with enzymes present in papaya latex, which might be used in peptide mapping, integral membrane protein solubility, preparation of Fab, screening of antibody, in the treatment of herniated lumber intervertbral disc, production of non-steroidal anti-inflamatory drug (NSAIDs) etc.

## Materials and methods

*Carica papaya* latex was collected from unripe fruits of female papaya tree grown in natural habitat following National Biodiversity Authority guidelines. Chlorauric acid (HAuCl4) was purchased from Sigma (Bangalore, India). Cell lines were procured from National Centre for Cell Sciences (Pune, India).

### Collection of papaya latex

Latex was collected by making several longitudinal incisions on the surface of the unripe papaya fruits. The collected latex was stored at -20 °C for further experiment. The latex was further diluted with water (1:1 ratio), and centrifuged at 5000×*g* at 4 °C for 10 min. The supernatant constituting aqueous fraction (AF) was separated from the pellet and was used for the synthesis of nanozymes.

### Protein estimation of AF of papaya latex

Protein estimation of AF was done by following Bradford method.

### Synthesis of gold nanozymes

Various parameters such as concentration of AF, concentration of HAuCl_4_ aqueous solution and reaction time were optimized (Table No. 1). Concentration of AF was optimized by varying its volume (1–10%, v/v) against the fixed 0.75 mM concentration of HAuCl_4_ aqueous solution. For synthesis of prismatic nanozymes, 5% (v/v) of AF was mixed with 0.75 mM HAuCl_4_ aqueous solution. The final volume of the mixture was made up to 5 ml with double distilled water. The resultant solution was kept in a domestic microwave oven [900 W, 2.45 GHz, LG MO-MC-767 W/WS (LG Electronics, Pvt. Ltd., India)] and irradiated for 50 s. The concentration of HAuCl_4_ was optimized by reacting 5% (v/v) AF with varying concentration (0.5–1.25 mM) of HAuCl_4_ solution. Similarly, the reaction time for the synthesis of these specific nanozymes were optimized by incubating the reaction mixture of 5% (v/v) AF and 0.75 mM HAuCl_4_ aqueous solution for different time periods ranging from 30 to 70 s.

### Characterization of nanozymes

The surface plasmon resonance (SPR) properties of the synthesized nanozymes were studied by subjecting the product samples to UV–VIS spectrophotometer (Tecan, Model: Infinite M 200) at the wavelength between 200 and 800 nm. Morphological details of the nanozymes were analyzed by Scanning electron microscope (FESEM, SIGMA 300, ZEISS) and transmission electron microscope (TEM, JEOL 2100 UHR-TEM). The size distribution of the particle was determined by dynamic light scattering (DLS) analyzer (Anton Paar, Litesizer 500). X-ray diffraction (XRD) pattern of the nanozymes were studied on a Bruker D8 ADVANCE X-ray powder diffractrometer (Bruker AXS Inc.) by using Cuα (λ = 1.54A^°^ ) source in the region of 2ɵ from 30° to 75°. The coating of nanoprisms by latex protein was investigated by FTIR spectra on FT-IR spectrophotometer (Shimadzu-IR Affinity^−1^) in the range of 450–4000 cm^−1^. Adsorption of AF proteins on synthesized gold nanozymes was evaluated by “coagulation test”. The coagulation test was done by adding 2.5 ml of 10% NaCl solution dropwise to 25 ml of nanozyme solution under constant stirring for 30 min^59^. Thermal properties of biomolecules present in nanozymes were also studied with Thermo gravimetric analyzer (TGA) (Model: TGA-7, Perkin-Elmer).

### Confirmation of proteolytic activity of nanozymes

The proteolytic activity of nanozymes was confirmed by milk clot test^60,61^ as well as papain inhibition test by chicken egg white cystatine^62^. For milk clot test, briefly, diluted AF was added to sterile milk in the ratio of 1:10. Different concentrations of nanoprisms (0.5–8%, w/v) was prepared (sonicated) in 100 µl of doubled distilled water of which final volume was adjusted to 1 ml and mixed with sterile milk in the ratio of 1:10. Sterilized milk, The quantitative enzymatic activity was determined by following the standard formula (Milk Clotting activity (U/g)) = 100/Ct X S/E, Where, Ct = Clotting time, S = Final volume of the milk and E = Amount of nanozymes). GNPs synthesized by sodium citrate (chemical method) and aqueous extract of *Centella asiatica* (green method) was also used for the test as control group. Synthesis of GNPs by sodium citrate and *Centella asiatica* was done by following the standard procedure as reported earlier^63^. For papain inhibition test, 1 ml of chicken egg white solution was added to the three different reaction mixtures (AF; sterile milk and AF; sterile milk and synthesized nanoprisms respectively). The chicken egg white solution was prepared by mixing lyophilized egg white in double distilled water (w/v) and was added in the ratio of 1:1 for all reaction mixtures. The whole test was performed under sterile condition at 40 °C for 72 h.

### Cytotoxicity studies

L929 (Rat fibroblast) and HeLa (Human cervical cancer) cell lines were used to study the cytotoxicity of nanozymes. Both cell lines were procured from National Centre for Cell Science (Pune, India) and were maintained by following the guidelines of the supplier. Cytotoxicity of the crude latex and synthesized nanozymes was studied by the MTT (3-[4,5-dimethylthiazole-2-yl]-2,5-diphenyl tetrazolium bromide) assay.

Cell viability was calculated according to the following formula:

$$\mathrm{Cell\,\, viability }(\mathrm{\%}) =\mathrm{ Nt}/\mathrm{Nc}$$

where Nt and Nc are the mean absorbance of GNPs treated and untreated cells, respectively.

### In vivo acute oral toxicity study

Swiss albino female mice were procured from authorized supplier and maintained under veterinary supervision following Institutional Animal Ethics Committee (IAEC) and Committee for the purpose of Control and Supervision of Experiments on Animals (CPCSEA) guidelines for the present experiment. The experiment was conducted following OECD guideline 423 for acute oral toxicity and was approved by the Institutional Animal Ethics Committee, Assam Agricultural University, Khanapara, Assam with Approval No. 770/ac/CPCSEA/FVSc/AAU/IAEC/14-15/278. The study is reported according to the ARRIVE guidelines. Here, 12 numbers of clinically healthy mice weighing 30–35 g were opted and divided randomly into 4 different groups; each consisting of three.5% carboxymethylcellulose (CMC) solution was used as a vehicle for homogenous dispersion of gold nanozymes and easier administration. Animals in group 1, 2 and 3 received single oral dose starting from 2000 mg, 1000 mg and 300 mg per kg body weight respectively. In group 4, plain CMC solution was administered orally and maintained as control. Animals were observed for behavioral changes and clinical abnormalities. Following CPCSEA and human method of sacrifice, histological Samples (pieces of kidney, liver and spleen) in 10% neutral buffer formalin (NBF) were collected after 14 days from the date of dosing for all groups. Samples were processed for Hematoxylin & Eosin (H&E) staining and cross-sectional images were taken at 400× magnification.

## Supplementary Information

Supplementary Information.

## Data Availability

All data generated or analysed during this study are included in this published article and its supplementary information files.
