# Supplementary material for: Papaya latex mediated synthesis of prism shaped proteolytic gold nanozymes
Source: Sci Rep. 2023 Apr 12;13:5965. doi: 10.1038/s41598-023-32409-7 (PMC10097869; doi:10.1038/s41598-023-32409-7)
Supplement: Supplementary file 1 — Supplementary Information. [file 41598_2023_32409_MOESM1_ESM.docx]

**Papaya latex mediated synthesis of prism shaped proteolytic gold nanozymes**

*Ajoy Kumar Das^1,2^, Jon Jyoti Kalita^2^, Maina Borah^3^, Suradip Das^2^, Manav Sharma^2^, , Dhiren Saharia^4^, Kushal Konwar Sarma^5^, Samrat Bora^1^ and Utpal Bora^2^

^1^Department of Botany, Arya Vidyapeeth College, Gopinath Nagar, Guwahati- 781 016, Assam, India

^2^Department of Biosciences and Bioengineering, Indian Institute of Technology, Guwahati, Assam, India

^3^Department of Botany, Pandu College, Pandu, Guwahati- 781 012, Assam, India

^4^ Saharia’s Path Lab and Blood Bank, Guwahati- 781 005, Assam, India

^5^Department of Surgery and Radiology, College of Veterinary Sciences, Assam Agriculture University Campus, Khanapara, Guwahati -781 022, Assam, India.

**Supplementary material**

Protein estimation of AF (aqueous fraction) was done by following Bradford and SDS-PAGE method (Figure 1). Concentration of AF was optimized by varying its volume (1-10%, v/v) against the fixed 0.75 mM concentration of HAuCl_4_ aqueous solution. For synthesis of prismatic nanozymes, 5% (v/v) of AF was mixed with 0.75 mM HAuCl_4_ aqueous solution. The final volume of the mixture was made up to 5ml with double distilled water. The resultant solution was kept in a domestic microwave oven [900W, 2.45 GHz, LG MO-MC-767 W/WS (LG Electronics, Pvt. Ltd., India)] and irradiated for 50 seconds. The concentration of HAuCl_4_ was optimized by reacting 5% (v/v) AF with varying concentration (0.5-1.25 mM) of HAuCl_4_ solution. Similarly, the reaction time for the synthesis of these specific nanozymes were optimized by incubating the reaction mixture of 5% (v/v) AF and 0.75 mM HAuCl_4_ aqueous solution for different time periods ranging from 30-70 seconds.

The surface plasmon resonance (SPR) properties of the synthesized nanozymes were studied by subjecting the product samples to UV-VIS spectrophotometer (Tecan, Model: Infinite M 200) at the wavelength between 200 to 800 nm.

The synthesis of gold nanozymes was visually confirmed by observing the changes of colour of the reaction mixture from yellow to ruby red. Formations of nanozymes were determined by UV-VIS spectral analysis of the coloured mixture. Nanozymes synthesized with 5% AF (v/v) and 0.75 mM HAuCl_4_ exhibited intense and narrow SPR peak centered at around 555 nm (Figure 2A).

UV-VIS spectra of the reaction mixture prepared with 5% of AF (v/v) and varying concentration of HAuCl_4_ (0.25 to 1.25mM) showed an intense and narrow SPR peak at 550 nm for 0.75 mM of HAuCl_4_ (Figure 2B).

Optimization of the reaction time was done by synthesizing nanozymes at different irradiation time (30-70 seconds) with optimized concentration of HAuCl_4_ (0.75 mM) and AF (5%, v/v). The SPR peak of the reaction mixture suggested the enhancement of nanozymes synthesis from 35-50 seconds (Figure 2C).

The UV-VIS spectra were taken to check the stability of the synthesized nanozymes which was stored for 90 days at 4º C after 15 minutes of sonication before taking the reading. No variation was seen in the spectra for both freshly synthesized nanozymes and stored nanozymes (Figure 3). This indicates that the synthesized nanozymes was found stable till the end of the experiment.

The quantitative enzymatic activity was determined according to the formula (MCA= 100/CT(s) X S/E, where, CT= Clotting Time, S=Final Volume of the Milk and E= Amount of Nanozymes ) suggested by Silva et al, 2005 with slight modification. The MCA (Milk Clotting activity) value was ranged from 5.208 (U/g) to 14.705 (U/g) (Table 1).

**Supplementary Tables**

**Supplementary Table -1 Enzymatic activity of different concentrations of nanozymes in milk clot assay**

| **Percentage of Nanozymes** | **Amount of Nanozymes**  **( in Gram)** | **Clotting Time (in Seconds)** | **MCA value**  **(in U/g)** |
| --- | --- | --- | --- |
| 0.5 | 0.0005 | No Clotting | - |
| 1.0 | 0.001 | 61200 | 14.705 |
| 2.0 | 0.002 | 43200 | 10.416 |
| 4.0 | 0.004 | 28800 | 7.812 |
| 6.0 | 0.006 | 25200 | 5.952 |
| 8.0 | 0.008 | 21600 | 5.208 |

**Supplementary Figures**


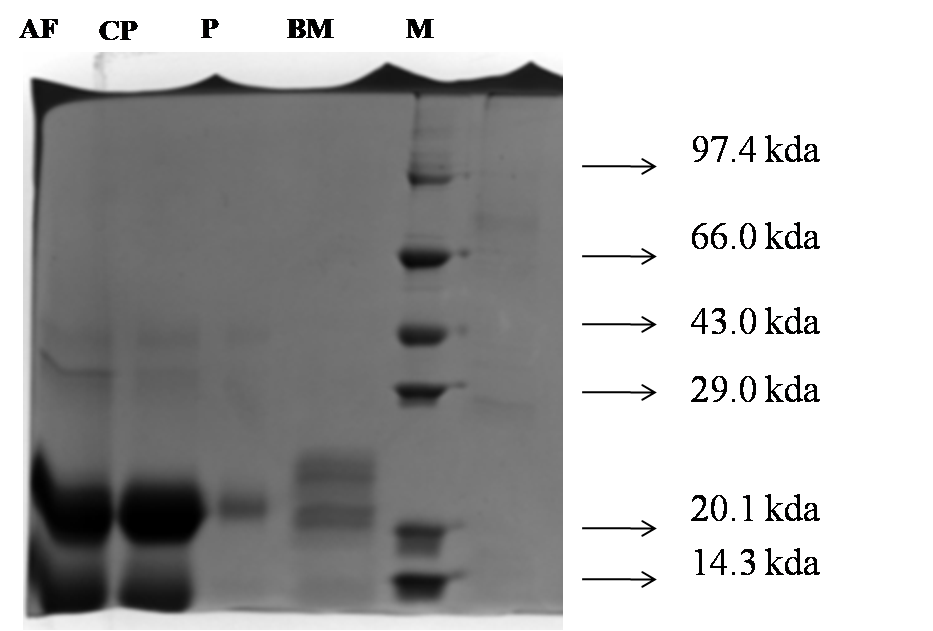


**Supplementary Figure 1**


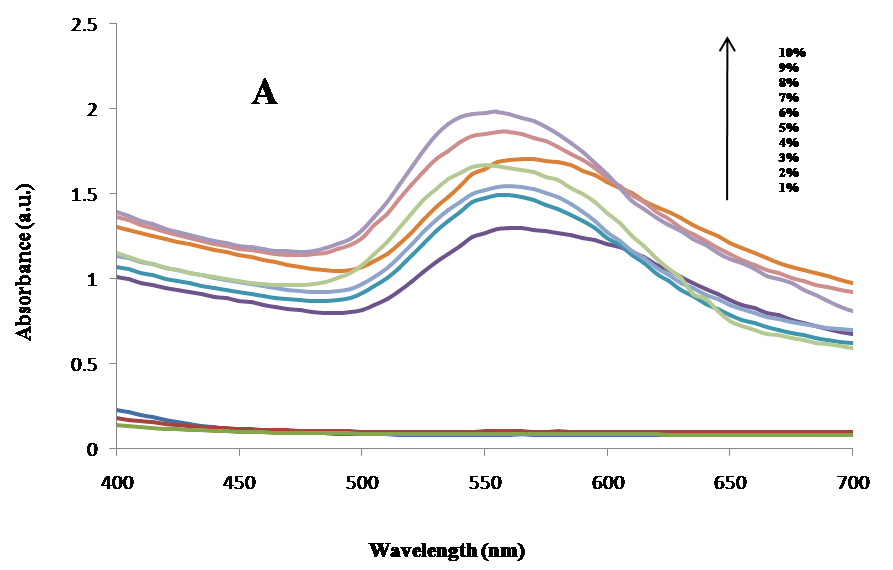


**Supplementary Figure 2A**


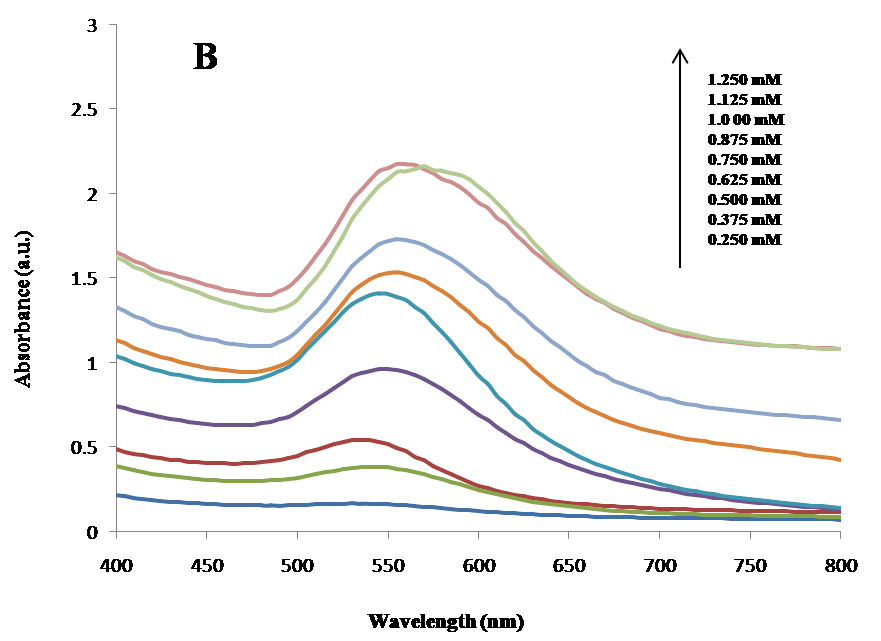


**Supplementary Figure 2B**


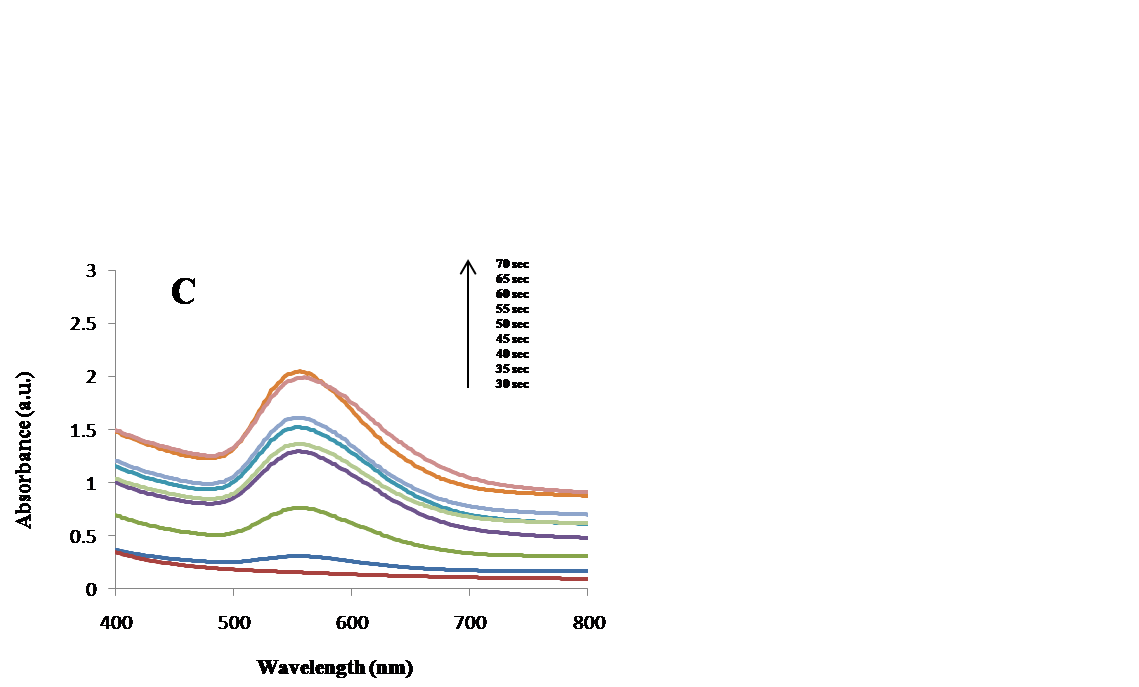


**Supplementary Figure 2C**

**
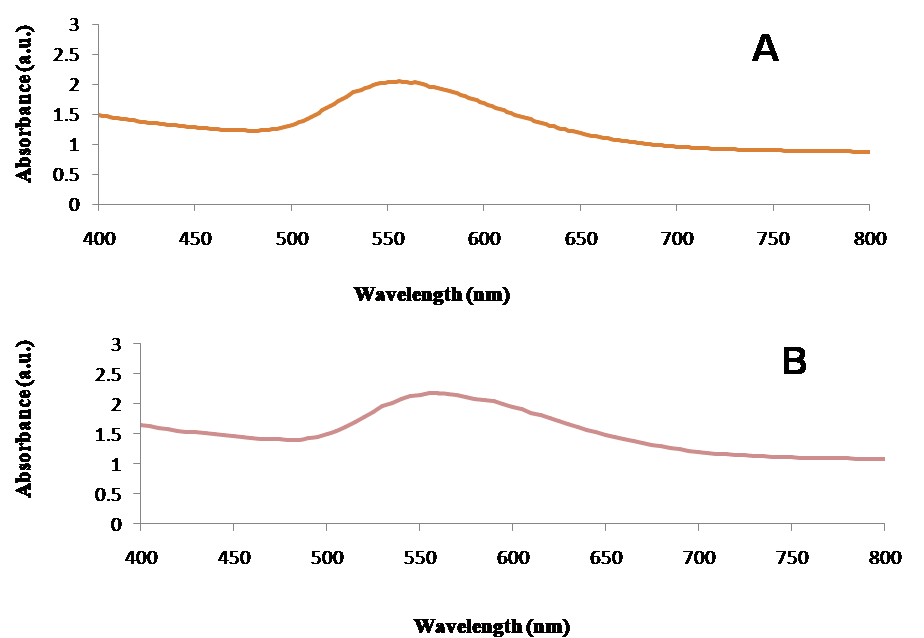
**

**Supplementary Figure 3**

**Supporting Information**

**Figure 1. SDS-PAGE analysis of *Carica papaya* L. latex where, AF= Aquous fraction, CP= Crude papain, P= Papain BM= Bromalin, M= Marker (TIF)**

**Figure 2. A= UV-VIS spectra of GNPs synthesized by reacting 0.75 mM HAuCl_4_ aquous solution with different volume fractions (1 – 10%) of AF; B= UV-VIS spectra of GNPs synthesized by reacting 5% of AF with 0.75 mM HAuCl_4_ aquous solution; C= UV-VIS spectra of GNPs synthesized by reacting 5% of AF with 0.75 mM HAuCl_4_ aquous solution for different time periods (30 – 70 secs) (TIF)**

**Figure 3. A= UV-VIS spectra of GNPs synthesized freshly by Optimized condition B= UV-VIS spectra of GNPs (Sonicated for 15 minutes before taking reading) that stored for 90 days at 4 ºC (TIF)**

**Reference:**

Silva, S.V. & Malcata, F.X.Studies pertaining to coagulant & proteolytic activities of plant protease from *Cynara candunculata*. *Food Chem*. 89, 19-26 (2005).
